# Unraveling the Deep Genetic Architecture for Seedlessness in Grapevine and the Development and Validation of a New Set of Markers for *VviAGL11*-Based Gene-Assisted Selection

**DOI:** 10.3390/genes11020151

**Published:** 2020-01-30

**Authors:** Nallatt Ocarez, Nicolás Jiménez, Reynaldo Núñez, Rocco Perniola, Antonio Domenico Marsico, Maria Francesca Cardone, Carlo Bergamini, Nilo Mejía

**Affiliations:** 1 Instituto de Investigaciones Agropecuarias (INIA), Centro Regional de Investigación La Platina, Santiago RM 8831314, Chile; nallatt.ocarez@gmail.com (N.O.); npjimene@gmail.com (N.J.); reynaldo.ns@gmail.com (R.N.); 2 Consiglio per la ricerca in agricoltura e l’analisi dell’economia agraria (CREA), Centro di ricerca Viticoltura ed Enologia, 70010 Sede di Turi (BA), Italy; rocco.perniola@crea.gov.it (R.P.); adomenico.marsico@crea.gov.it (A.D.M.); mariafrancesca.cardone@crea.gov.it (M.F.C.); carlo.bergamini@crea.gov.it (C.B.)

**Keywords:** seedlessness, stenospermocarpy, QTLs, SNPs, SSRs, gene-assisted selection, *VviAGL11*

## Abstract

Seedless inheritance has been considered a quasi-monogenic trait based on the *VvAGL11* gene. An intragenic simple sequence repeat (SSR) marker, p3_VvAGL11, is currently used to opportunely discard seeded progeny, which represents up to 50% of seedlings to be established in the field. However, the rate of false positives remains significant, and this lack of accuracy might be due to a more complex genetic architecture, some intrinsic flaws of p3_VvAGL11, or potential recombination events between p3_VvAGL11 and the causal SNP located in the coding region. The purpose of this study was to update the genetic architecture of this trait in order to better understand its implications in breeding strategies. A total of 573 F1 individuals that segregate for seedlessness were genotyped with a 20K SNP chip and characterized phenotypically during four seasons for a fine QTL mapping analysis. Based on the molecular diversity of p3_VvAGL11 alleles, we redesigned this marker, and based on the causal SNP, we developed a qPCR-HRM marker for high-throughput and a Tetra-ARMS-PCR for simple predictive analyses. Up to 10 new QTLs were identified that describe the complex nature of seedlessness, corresponding to small but stable effects. The positive predictive value, based on *VvAGL11* alone (0.647), was improved up to 0.814 when adding three small-effect QTLs in a multi-QTL additive model as a proof of concept. The new SSR, 5U_VviAGL11, is more informative and robust, and easier to analyze. However, we demonstrated that the association can be lost by intragenic recombination and that the e7_VviAGL11 SNP-based marker is thus more reliable and decreases the occurrence of false positives. This study highlights the bases of prediction failure based solely on a major gene and a reduced set of candidate genes, in addition to opportunities for molecular breeding following further and larger validation studies.

## 1. Introduction

Table grapes are a consumable product developed by breeding activities that deal with global demands coming from producers, plant nurseries, and consumers [1]. Additionally, significant pressure comes from new environmental conditions [2], from the requirement of more environmentally-friendly practices for a sustainable viticulture [3,4], and trends [5]. The muscle in grape breeding is the generation of a large number of seedlings derived from multiple crosses, and the brain is the delicate art of selecting promissory lines bearing a minimum list of desirable traits. From a practical perspective, some breeders consider that one in a thousand individuals deserves to be evaluated twice, and one in ten thousand has the potential to become a new variety. Selection, based on a phenotypic evaluation of traits such as berry size, berry skin color, and bunch size, is simple but requires the plant to achieve maturity (2–3 years after crosses were made) [6]. However, for other complex or more subjective traits such as crunchiness, flavor, and acidity, evaluations require sensorial and/or specific instrumental analysis and, in addition to mature seedlings, also require phenotype stability, which can take an additional couple of seasons to achieve. For any given case of simple or subjective traits, the main issue is the number of seedlings that have to be established in the field and evaluated in the short period of time associated with seasonal berry ripening. Assisted selection, based on DNA molecular markers, can be applied from the very early stages of seedling development and with total independence from the fruit developmental and ripening process [7]. This approach is currently being applied in several breeding programs, especially for quasi-monogenic traits that are subject to negative selection, such as seedlessness or fungal disease resistance, through discarding of undesirable plants.

Seedlessness, in particular, is one of the most popular desired traits among consumers [1]. With high heritability and the dominance of the seedless allele over a large number of seeded alleles, the stenospermocarpic seedlessness [8] of table grapes is ideal for both breeding and application of assisted selection. Generally, homozygous seedless genotypes result in very small berries (<10 mm of equatorial diameter) not suitable for the table grape industry that prefers larger berries (>16 mm). For this reason, most of the seedless varieties are heterozygous, i.e., they carry the *VviAGL11* seedless allele in combination with any of the other seeded alleles; however, the degree of seedlessness (from very unnoticeable seed traces to large and lignified rudiments) might depend on the associated *VviAGL11* seeded allele and from additional minor genes. Some seeded alleles are apparently more prone to domination by the seedless allele [9] or additional minor genes [10] with effect over the phenotype are co-inherited with particular seeded alleles. Both the genetic and molecular bases of the phenotypic dominance of the seedless allele and the existence of minor effect genes remain largely unknown.

An intragenic microsatellite (simple sequence repeat, SSR) marker mapped on the regulatory region of the *VviAGL11* candidate gene, called p3_VvAGL11, was developed by Mejía et al. [9]. Its usefulness was validated independently by Bergamini et al. [11], who tested the marker and highlighted it as an effective tool for early selection of seedlessness, although some false positives (~2%) were occasionally reported over the years by breeders in Ukraine, the United States, and Italy (personal communications). For assisted selection purposes, the genotyping pipeline should be simple, fast, and low cost, without compromising information content. In principle, SSR genotyping is a standard technique based on PCR and amplicon size analysis by electrophoresis (polyacrylamide gel electrophoresis or capillary electrophoresis). However, inconsistency in terms of results make it difficult to compare data among laboratories: the large variety of fragment analyzers with different migration rates, dyes, and software affect result consistency. Within the same laboratory, amplicon migration can also be affected by the fluorescent dye (between 1 and 3 bp of difference [12], as well as by the inconsistent addition of 3′ A-overhangs to amplicons [13], complicating both the automatic allele calling and the analysis, especially when co-electrophoresis is used to reduce costs. Additionally, allele definition for p3_VvAGL11 SSR is related to size variation in PCR products, which should be correlated to differences in the number of repeats in the simple (GA) motif. However, size variation in this case is also due to two additional insertion-deletions mutations (INDELs) flanking the main SSR. For this reason, amplicons of the same size can, in reality, be different alleles and thus reduce the informativeness of the marker regarding diversity. Finally, but no less relevant, for the p3_VvAGL11 SSR marker, non-template PCR controls tend to amplify a PCR product that is very similar in size to that of the seedless allele, and this amplicon originates from genomic *E. coli* DNA [14] and results in false positive results, especially when using low-quality or -quantity grapevine genomic DNA as templates. Although the p3_VvAGL11 marker is widely and routinely applied in breeding programs and SSRs are economical to score, advances in technology are shifting toward single nucleotide polymorphisms (SNPs) because they are amenable to high-throughput methods and have lower genotyping error rates [15].

It has recently been reported [16] that a mutation (Arg-197-Leu substitution) in exon 7 of the coding sequence of the *VviAGL11* gene, previously identified and mistakenly reported as non-causal [9], is fully linked to stenospermocarpy. Silencing of the heterologous gene to *VviAGL11* in *Solanum lycopersicum* produces seedlessness [17], and the ectopic expression of *VviAGL11* in *Arabidopsis AGL11* mutant restores the wild type phenotype and confirms its role in seed morphogenesis [18]. Both works support, at a functional level, *VviAGL11* as the main gene responsible for seedlessness. The identification of false positive individuals (identified genetically as seedless but containing true seeds) based on the p3_VvAGL11 SSR marker [9] can be easily explained by the ancestral allele without the Arg-197-Leu mutation or if recombination events occurs between the promoter region and the coding region that harbors the mutation. Meanwhile, the identification of false negative individuals (identified genetically as seeded but containing only seed traces) can be caused by minor QTLs that still need to be identified and can be responsible for phenotypic variation within heterozygous genotypes. To date, a marker for assisted selection purposes based on the *VviAGL11* Arg-197-Leu mutation is not available for breeding purposes.

The present study is based on a large F1 mapping population (*n* = 573) derived from the cross of Muscat of Alexandria × Crimson Seedless (MA × CS) characterized at the phenotypic level for seedlessness over four seasons. We re-analyzed the genetic architecture of seedlessness using a fine QTL mapping strategy, confirming the major QTL that points at *VviAGL11* as mainly responsible for the genetic determinism of seedlessness in table grapes and revealing the existence of several significant and suggestive, yet stable, minor QTLs for seedlessness. For assisted selection purposes, we developed and validated a new set of *VviAGL11* SNP-based markers that can improve molecular selection because they are based on the Arg-197-Leu mutation and are simple, fast, and cost-efficient to apply. For the analysis of VviAGL11 diversity, we developed a new marker, mapped on the same region of SSR of p3_VvAGL11, that is more informative, reliable, and robust, and also easier to analyze. 

## 2. Materials and Methods

### 2.1. Plant Material 

For QTL mapping analysis, the mapping population was composed of 573 unique F1 individuals, and the cross was performed in 2010 between Muscat of Alexandria (MA, seeded maternal genotype) and Crimson Seedless (CS, seedless paternal genotype). The population was established in the field within a random block that included both parental genotypes, over its own roots, in 2012 at La Platina Regional Research Center (INIA, Santiago, Chile: 33°34′15.63″ S 70°37′45.03 O) and has been fruitful since 2014. The vines were trained in a quadrilateral cordon trellised on a double cross arm system and spaced 2 m (between vines) by 3 m (between rows). Agricultural management practices were carried out following recommendations from the local breeding program.

### 2.2. Seedlessness Phenotypic Evaluation for QTL Mapping 

**Figure 1:**
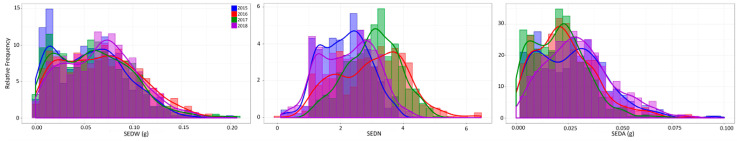
Distribution of the MA × CS progeny (*n* = 573) for SEDW, SEDN, and SEDA across four evaluated seasons. Graphs are presented as overlapped blue, red, green, and purple bar graphs for 2015, 2016, 2017, and 2018 seasons, respectively.

In this work, seedlessness was analyzed during four consecutive seasons (2015–2018) as seed fresh weight (SEDW) per berry, the number of seeds per berry (SEDN), and the average weight of one seed (SEDA) calculated as the ratio of SEDW/SEDN. For each single individual, a total of three clusters were randomly selected and harvested between 16 and 18 °Bx (36.5–37 in the modified Eichhorn-Lorenz system of berry development [19]). Twenty-five berries were randomly selected in each cluster to analyze SEDW and SEDN; all the seeds or seed traces were isolated and cleaned from pulp remains before counting and weighting them in an analytical balance with a readability range between 0.01 and 0.1 mg. An average between the three analyzed clusters was taken as phenotypic values for SEDW and SEDN in each season. Phenotypic data could not be normalized through common transformation algorithms due to severe deviation (bimodal distribution, especially for SEDW, Figure 1 and Supplementary Figure S3) from normality in all seed phenotypic subtraits. Histograms and trend lines were prepared with ggplot2 version 3.2.1 for R version 3.6.2.

QTL analysis with deviations from normality is not affected by such deviations according to Van Ooijen [20]. A non-parametric Spearman correlation between seasons and traits was calculated with a two-tailed *p*-value (*p* < 0.05) in Prism 8.1.2 software. The raw phenotypic values were used to estimate (Best Linear Unbiased Predictors) BLUPs through InfoStat/E (v2018e) [21]. An analysis of variance under a mixed model with a random genotype effect and a fixed block effect was performed by using restricted maximum likelihood (REML) for variance component estimation in which each season was considered as a different environment.

### 2.3. Seedlessness Phenotypic Evaluation for Validation Purposes 

For validation purposes, several F1 progenies were used: the large (*n* = 573) MA × CS progeny (seeded × seedless) and two small (seeded × seedless) progenies (*n* = 48), from the CREA breeding program were used: Red Globe (RG) × Autumn Royal Seedless (AR) and Red Globe (RG) × Regal Seedless (RS). Additionally, a group of individuals composed of 174 F1 hybrid plants derived from 55 different crosses of the CREA breeding program. Moreover, a set of 209 cultivars that included the 100 varieties that represent 53% of the total diversity and 99% of the restricted diversity (alleles with frequencies > 0.05 %) of the CREA collection [11].

For RG × AR and RG × RS progenies held by CREA seedless phenotyping were considered suitable for procedures similar to MA × CS, but a total of 15 berries was considered for each seedling. The germplasm collection and hybrids from the CREA breeding program were phenotyped for seedlessness as described by Bergamini et al. [11]. A quali-quantitative value was also established to fit a binary distribution to describe seedlessness: 0.045 g is the average of seed fresh weight that was defined as the cutoff value between seedless and seeded. Veracity of the phenotype was checked in the VIVC database (http://www.vivc.de/), the European database (http://www.eu-vitis.de/), or the database from the Centre de Resources Biologiques de la Vigne (Collection ampélographique de Vassal-Montpellier (https://bioweb.supagro.inra.fr/collections_vigne/Home.php).

### 2.4. Genetic Characterization of MA × CS by Illumina Vitis 20k SNP Chip Genotyping

Prior to the genotyping of the MA × CS progeny with the *Vitis* 20K SNP chip (https://urgi.versailles.inra.fr/Species/Vitis/GrapeReSeq_Illumina_20K), the progeny was authenticated to discard self-pollinations or non-expected outcrosses. Authentication was performed genotyping a larger progeny with fully informative SSRs VVMD21 [22], VVS2 [23], and p3_VvAGL11 [9]. In the MA × CS progeny, these markers segregate (211/221 × 204/204), (158/174 × 160/176), and (173/188 × 186/196), respectively. SSR genotyping by PCR amplifications were performed in a 20 μL reaction mixture containing a 0.25 µM of each labeled primer (forward primers of VVMD21, VVS2, and p3_VvAGL11 with FAM, VIC, and NED respectively), 0.4 mM of each dNTP, 1.5 mM MgCl_2_, 0.25 U Taq polymerase, 5 ng of template DNA, 0.2 mM Red Cresol, and 12% sucrose. A Veriti thermal cycler (Applied Biosystems, Foster City, CA, USA) was programmed as follows for PCR amplification: an initial denaturation (2 min at 95 °C) followed by 35 cycles of denaturation (30 s at 95 °C), annealing (30 s at 60 °C), and extension (10 s at 72 °C), and a fill-in step of 4 min at 72 °C was added at the end. SSR PCR products, labeled with fluorescent dyes, were resolved by capillary co-electrophoresis according to standard procedures recommended for the ABI 3130xl Genetic Analyzer (Applied Biosystems, Foster City, CA, USA). Data were analyzed with GeneMapper 4.0 (Applied Biosystems, Foster City, CA, USA). Only authenticated and fruitful progeny were considered for further analyses.

Genotyping with the Illumina 20K SNP Infinium chip (18,071 SNP markers [24]) was performed at IntegraGen (Evry, France). Polymorphic markers and non-severely distorted markers (6,221 SNPs, 34.4%) were filtered down to 5418 using only high quality SNPs flagged “AUX=1” in the cluster file GrapeReSeq_SNP Table_all_180413.xlsx available at https://urgi.versailles.inra.fr/content/download/2688/23435/file/GrapeReSeq_SNP%20Table_all_180413.xlsx. Each one of the 18,071 SNP markers were renamed according to their actual position in the 12X.2 Assembly version of the grapevine reference genome sequence from The French-Italian Public Consortium (PN40024 [25]). The position was defined by a local blast of flanking SNP sequences against the reference genome. 

Genomic DNA of the mapping progeny and other validation progenies, a germplasm collection, and hybrids from the CREA breeding program were extracted, after the homogenization of 100 mg of young leaves with a tissue homogenizer and following the manufacturer’s instructions of the DNeasy Plant Mini Kit (Qiagen, Germany). The concentration was measured with the Qubit 2.0 fluorometer (Invitrogen, CA), and the quality of the DNA was checked by spectrophotometry and electrophoresis prior to being used as a template for genotyping. For comparative purposes, DNA was also extracted from particular genotypes with a protocol described by Lodhi et al. [26] and with a protocol to obtain a crude extract described by Xin et al. [27] in order to test the reliability and robustness of developed markers with different quality templates.

### 2.5. Fine QTL Analysis of Seedless Subtraits in Muscat of Alexandria × Crimson Seedless F1 Mapping Population 

Genetic maps were built using 1541 SNPs that correspond to unique bins segregating from Muscat of Alexandria and 1534 SNPs that correspond to unique bins segregating from Crimson Seedless. Genetic maps for QTL analysis have a coverage of 1575 and 1430 cM in MA and CS, respectively, resulting in an average distance of 313 kb between SNPs (1.0 cM between SNPs). The maximum likelihood mapping algorithm with Haldane’s mapping function implemented in JoinMap 4.1 [28] was used with defaults settings for mapping analysis. The Fixed Order option was used considering the updated position of every SNP of the 20K chip. Parental linkage maps were built using the double haploid configuration. Phases were automatically determined by JoinMap 4.1. Maps and QTLs were drawn using the software MapChart 2.2 [29]. Best linear unbiased predictor (BLUP) values, estimated on the base of four seasons, were used for QTL mapping. The regression algorithm was used for all QTL analysis. A preliminary QTL analysis was performed in both parental maps using interval mapping (using the regression approach algorithm) and Kruskal–Wallis, both included in MapQTL 6.0 [20]. For each putative QTL, the closest markers to the peak of the LOD profile were tested using the automatic cofactor selection procedure. Markers accepted as co-factors where then used to perform a multiple QTL mapping (MQM) test and to determine the total phenotypic variation explained by the QTL. A permutation test (10,000 permutations, with a genome-wise and chromosome-wise type error rate of 0.05) was used to establish the threshold level at which a QTL was declared significant or suggestive according to Churchill and Doerge ([30]) and Doerge and Churchill [30,31]. QTLs were established as significant or suggestive when the detected LOD was higher than the threshold LOD for a genome-wise or chromosome-wise type error, respectively. The stability of QTLs was estimated with the defined cofactors performing the MQM analysis for each individual season using phenotypic values. One-LOD and two-LOD support confidence intervals were constructed for each QTL according to [32] and represented over genetic parental maps.

### 2.6. Identification of Putative Candidate Genes within QTL Confidence Intervals

Identified confidence intervals, with 1-LOD support, directly define the specific genomic segments in which to search for candidate genes, since the name of each SNP marker corresponds to its actual position in the reference genome assembly V12X.2. To define the possible candidate genes within such intervals, we used the structural and functional annotations of both CRIBI V2.1 (http://genomes.cribi.unipd.it/DATA/V2/V2.1/) and VCost V3 [33].

### 2.7. Evaluation of Multi-QTL Predictive Model to Increase Assisted Selection Efficiency

Considering the additive effect of QTLs (that segregate independently or are separated by more than 50 cM), we developed a simple predictive model that includes the simultaneous evaluation of multiple QTLs, represented by the multiple SNPs used as cofactors in the multiple QTL mapping analysis. QTLs were selected methodically and added one by one according to their individual phenotypic effect (the explained variance calculated by the MQM analysis) in decreasing order. Thus, for a single marker, two genotypes exist (due to the ploidy level of the organism), a or b, with a favorable or unfavorable effect over seedlessness. For n markers, 2^n^ genotypes or factors exist, and the most favorable combination of alleles was called an ideotype, which was determined by concatenation of the favorable individual genotypes. The phenotype and combined genotypes or haplotypes were tested by a one-way ANOVA (Minitab 17) without assuming equal variances.

### 2.8. Sequencing of VviAGL11 Regulatory and 5′UTR Regions for Variant Discovery and the Design of a New SSR For Assisted Selection

Based on a preliminary diversity analysis with the SSR p3_VvAGL11 of a collection of breeding material from INIA and CREA, we defined the existence of at least 10 *VviAGL11* alleles at the promoter level, including the reference allele from Pinot Noir PN40024, the most common seeded allele (186 bp), and the unique seedless allele (196 bp) described earlier [9]. For each allele, a 3 kb regulatory region of *VviAGL11* was isolated using the primers pVviAGL11-F and pVviAGL11-R described previously [17]. The PCR was performed using the Phusion High-Fidelity DNA Polymerase (New England Biolabs) following the manufacturer’s instructions. A-overhangs were added according to the instruction manual for the Gateway entry vector pCR8/GW/TOPO TA (Applied Biosystems, Foster City, CA, USA) to enable ligation of the PCR product in this vector. Colonies with the expected insert size were genotyped using SSR p3_VvAGL11 [9] to select clones with the expected variants. Between two and three clones per variant were sequenced by the Macrogen sequencing service (Korea) using the universal primers M13F-pUC (−40) and M13R-pUC (−40) and four internal oligos—AG_sq_01, AG_sq_06, AG_sq_08, and AG_sq_10 (described in [9])—to achieve a minimum of twofold coverage for the entire insert. Sequences were analyzed using Geneious 7.0.6 [34] and curated by hand. SSR p3_VvAGL11 contains two repeated (GA)n tracts. To design a new SSR suitable for assisted selection purposes, we included a third (GA)n repeat in the surrounding of p3_VvAGL11. The new SSR located in the 5′UTR was named 5U_VviAGL11 and amplifies fragments between 267 and 325 bp, where the seedless allele corresponds to 319 bp (337 with the M13 tail, Figure S1). Designed 5U_VviAGL11 forward and reverse oligos are 5′-CGC CCA TTC TCT CTC GCT AT-3′ and 5′-GTG CAA AAA CGC GTA TCC CA-3′, respectively. To reduce the costs of analysis, we also used the M13-tailed SSR with a sequence that contains a fusion of leading M13(−21) universal sequence (18 bp) with the forward described primer sequence—5′-TGT AAA ACG ACG GCC AGT CGC CCA TTC TCT CTC GCT AT-3′—and a fluorescently-labeled M13(−21) universal primer—5′-FAM/VIC/NED/PET-TGT AAA ACG ACG GCC AGT-3′—as described by Schuelke [35].

### 2.9. PCR Validation Assays of SSR 5U_VviAGL11

Validation assays included the comparison with SSR p3_VvAGL11 that also included M13-tailed SSR with the forward primer 5′-TGT AAA ACG ACG GCC AGT CTC CCT TTC CCT CTC CCT CT-3′, the reverse primer 5′-AAA CGC GTA TCC CAA TGA AG-3′, and the M13(−21) universal primer as described above. As a template, we used 10 ng of genomic DNA from all the material described above. The cycling profile was an initial denaturation step at 95 °C for 2 min, 27 cycles of denaturation for 30 s at 95 °C, annealing for 45 s at 60 °C, and an extension for 15 s at 72 °C, followed by 18 cycles of denaturation for 30 s at 95 °C, annealing for 45 s at 53 °C, an extension for 15 s at 72 °C, and a final extension for 10 min at 72 °C. PCR amplifications were carried out on a final volume of 20 µL containing 0.25 µM of M13(−21) universal fluorescently labeled primer, 0.25 µM of reverse specific primer, and 0.0625 µM of M13-tailed forward primer. PCR also contained 400 µM dNTPs, 1.5 mM MgCl_2_, 1.25 U of homemade Taq polymerase [36], and a 1X standard reaction buffer (50 KCl, 10 Tris-HCl, pH 8.8). PCR was performed in a Veriti 96-well Thermal Cycler (Applied Biosystems, Foster City, CA, USA). Amplicons were resolved by co-electrophoresis on an 3500xl Fragment Analyzer (Applied Biosystems, Foster City, CA, USA) injecting 2 µL of a mixture of PCRs that contains fluorescently labeled products in the following proportions: 1:1:1.5:2 and 2:2:3:4 for FAM/VIC/NED/PET-labeled products for p3_VvAGL11 and 5U_VviAGL11, respectively. For visualization, analysis, and automatic allele calling, we used GeneMapper Software v4.0 (Applied Biosystems, Foster City, CA, USA). To assess the informativeness of 5U_VviAGL11 SSR in other *Vitis* species, we also tested these markers in nine different species and four other members of the Vitaceae family.

### 2.10. Real-Time PCR and High-Resolution Melt Analysis for Rapid Detection of the Substitution Arg-197-Leu in the Seedless Allele of VviAGL11

A qPCR-HRM SNP marker was designed for the detection of the substitution Arg-197-Leu in the *VviAGL11* gene (Figure S2 and Table S1). The SNP [G/T] marker was named e7_VviAGL11 (named VvAGL11_e7 for an SSCP-based marker in [9]) and consists of the primer pair e7_VviAGL11_F 5′-TCA ATC ATA TTG GGC TGA AAG AAA TTG-3′ and e7_VviAGL11_R 5′-GCC ATC CAG GCA TTA GTT TCT C-3′, which produces an amplicon of 50 bp. As a template, we used 10 ng of genomic DNA from all the material described above. PCR and post-PCR high-resolution melting analysis were performed in a StepOne Plus Real-Time PCR System (Applied Biosystems, Foster City, CA, USA) using the KAPA HRM FAST qPCR Master Mix (KAPABIOSYSTEMS, Cape Town, South Africa) as follows. For a 20 µL final volume reaction, 10 µL of the Master Mix, 3.5 mM MgCl_2_, and a 0.25 µM concentration of each primer were combined. The qPCR thermal cycler was programmed with an initial denaturation (3 min at 95 °C) followed by 40 cycles of denaturation (5 s at 95 °C) and an annealing/extension (30 s at 62 °C). A dissociation curve was obtained during denaturing (15 s at 95 °C), heteroduplex formation (1 min at 40 °C and 15 s at 62 °C) and dissociation analysis, whereby the temperature was incremented at a ramp of 0.3%, with data acquisition between 62 and 95 °C. Variant calling was performed automatically with High Resolution Melt 3.1 software (Applied Biosystems, Foster City, CA, USA).

For high-throughput and economical implementation of assisted selection, a fast extraction and amplification method was coupled to the qPCR-HRM analysis based on the e7_VviAGL11 marker. The method described originally by Xin et al. [27] was adapted for the grapevine: ~15 mm^2^ of the sample coming from young leaves (fully expanded and without ribs) was supplemented with 50 µL of Buffer A (100 mM NaOH and 2% Tween 20 prepared just before use from 10 M NaOH and 20% Tween 20 stock solutions) and incubated at room temperature for 3 min after vortexing for 1 s (incubation at 95 °C for 10 min, as originally described, increments the release of inhibitors). Fifty microliters of Buffer B (100 mM Tris-HCl, 2 mM EDTA) was added by pipetting to slowly stabilize the solution mixing. The extraction was diluted to 2:25, and 5 µL of the dilution was used as template DNA for the qPCR-HRM described above. The Master Mix was supplemented with 0.01% (w/v) of non-acetylated UltraPure BSA (Ambion) and 1% of PVP-40. The PCR was performed as described above. The method was compared to the commercial protocol of the DNA Extract All Reagents Kit (Applied Biosystems, Foster City, CA, USA) that was used with the protocol indicated above. This kit-based extraction was diluted to 2:25, and 2.5 µL of the dilution was used as a template (larger amounts of template increased PCR inhibition). Reproducibility of this fast extraction and amplification method was tested with several dozens of samples, but we present results for representative genotypes only.

### 2.11. Tetra-Primer Amplification Refractory Mutation System PCR for Non-Sophisticated Detection of the Seedless Allele

The tetra-primer amplification refractory mutation system PCR (Tetra-ARMS PCR) is a simple, rapid, reproducible, and accurate method for assaying SNPs based on a single PCR amplification followed by electrophoresis resolution for variants determination. For e7_VviAGL11 SNP [G/T], the method amplifies both the seedless and any seeded allele, together with an internal control fragment (with respect to missed amplification). The region flanking the e7_VviAGL11 SNP was amplified by two outer primers that are also used with both allele-specific inner primers that were designed in an opposite orientation and for combination with the outer primers (Supplementary Figure S2 and Table S1). The specificity of the allele-specific primers is conferred by the specific match to the terminal 3′ nucleotide (with the seedless [T] or any seeded [G] allele) and the deliberate introduction of a mismatch at the −3 position from the 3′-terminus as described by Ye et al. [37]. The e7_VviAGL11 SNP [G/T] is asymmetrically located with respect to the common primers; thus, PCR products can be easily separated and differentiated by agarose (3%) gel electrophoresis. The Tetra-ARMS PCR for e7_VviAGL11 was performed in a 20 μL reaction mixture containing 0.15 µM of each of the following primers: the internal seeded specific and both external primers, and containing 0.60 µM of the internal seedless specific primer (Table S1), a 0.4 mM concentration of each dNTP, 1.5 mM MgCl_2_, 1 U of home-made Taq polymerase, 10 ng of template DNA, 0.2 mM Red Cresol, 12% sucrose, and 1.2X of NH4 PCR buffer (where a 1X standard NH4 reaction buffer contains 67 mM Tris-HCl at pH 8.8, 16 mM (NH4)2SO4, and Tween-20 0.01%). A Veriti thermal cycler (Applied Biosystems, Foster City, CA, USA) was programmed as follows for PCR amplification: an initial denaturation (2 min at 95 °C) followed by 35 cycles of denaturation (30 s at 95 °C), annealing (30 s at 58 °C), and extension (10 s at 72 °C), and a fill-in step of 4 min at 72 °C was added at the end. The reproducibility of this Tetra-ARMS PCR method was tested with several dozens of samples, but we present results for representative genotypes only.

### 2.12. Validation Assay: Comparison between SSR p3_VvAGL11, SNP e7_VviAGL11, and SSR 5U_VviAGL11

To validate the usefulness of new developed markers, in this work we analyzed the genotype–phenotype association using three groups of materials: (1) 573 F1 segregants of MA × CS (genotypes 173/188 × 186/196, where 196 is the seedless allele), 48 F1 segregants of RG × AR ((186/186) × (189/196)), and 48 F1 segregants of RG × RS ((186/186) × (186/196)); (2) 209 varieties, including the 100 varieties from the CREA germplasm collection reported by Bergamini et al. [11]; (3) 174 hybrids derived from 55 different crosses from the CREA breeding program [11]. Results obtained by SNP e7_VviAGL11 genotyping were compared with the most informative and already validated marker SSR p3_VvAGL11 [11]. The performance of the prediction capability for both markers was assessed, reducing the complexity of genotypes and phenotypes to a binary system (seeded and seedless categories as described in the phenotyping section) in order to fit a 2 × 2 contingency table containing true positive (TP), true negative (TN), false negative (FN), and false positive (FP) cases. Based on these descriptors, five measures were calculated according to [38]: sensitivity (true positive rate), specificity (true negative rate), precision (positive predictive value), negative predictive value, and accuracy. Derived from these measures, the false positive rate and false negative rate were calculated as described by Vihinen et al. [38]. In addition, to have a much more balanced evaluation of the prediction, the Matthews correlation coefficient (MMC) was calculated as described by Vihinen et al. [38] and Baldi et al. [39]. Statistical association between genotype, determined either by SSR 5U_VviAGL11 or by SNP e7_VviAGL11, and phenotype was tested from the 2 × 2 contingency table with Fisher’s exact test with a two-tailed *p*-value (https://www.graphpad.com/quickcalcs/contingency1/)

## 3. Results

### 3.1. Phenotypic Evaluations of the MA × CS Progeny Across Four Consecutive Seasons

**Figure 2:**
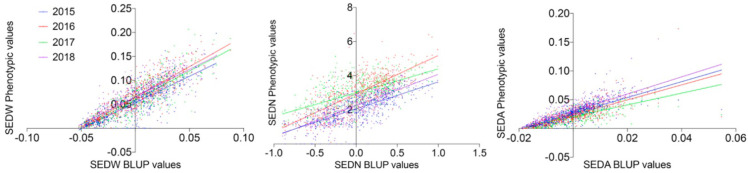
Correlation between phenotypic values for SEDW, SEDN, and SEDA with their respective best linear unbiased predictors (BLUPs). For all the four evaluated seasons (2015–2018), the average R2 was 0.7453, 0.5042 and 0.6204 for SEDW, SEDN, and SEDA, respectively, with *p* < 0.0001.

The MA × CS population (*n* = 573) revealed non-normal distributions for all three analyzed traits, none of which passed the Anderson–Darling normality test in any of the evaluated seasons (2015 to 2018 with *p* < 0.05). On average, skewness values were 0.402, −0.050, and 2.569, reflecting the approximately symmetric distribution for SEDW and SEDN and the highly skewed distribution for SEDA, respectively. The average of Kurtosis values was −0.288, −0.554, and 24.926. Seedlessness subtraits have a bimodal distribution (Figure 1 and Figure S3) rather than a normal distribution, which is explained by the effect of the seedless major loci. The distribution of SEDW, SEDN, and SEDA values across four seasons also showed a high interseason variation. For instance, SEDN presented on average one more seed in seasons in the years 2016 and 2017 than seasons in the years 2015 and 2018. This increase in seed number seems to be compensated for by the average seed size (SEDA), which was reduced in the years 2016 and 2017 (Figure 1). This degree of instability of the seedless phenotype is also common in varieties and advanced selections of breeding programs, which might be due to the partial dominance of the seedless allele when it is in a heterozygous state, like in most of the varieties. A correlation analysis between seasons for all the three evaluated traits showed an average correlation of 0.653, 0.3483, and 0.6183 for SEDW, SEDN, and SEDA, respectively (Supplementary Figure S4). The average of the phenotypic values does not reflect the described variability across seasons; thus, the best linear unbiased predictors (BLUPs) based on mixed models with random genetic effects were estimated on a per-genotype basis for QTL mapping and the estimation of their respective effects. Linear regression analysis showed a strong linear relationship between BLUPs and phenotypic means with an average R^2^ of 0.7453, 0.5042, and 0.6204 for SEDW, SEDN, and SEDA, respectively (Figure 2).

### 3.2. Genetic Map Construction for the MA × CS Progeny

Each parental linkage map covered more than 1500 cM with an average density of 1 cM between two adjacent SNPs, which corresponds on average to 302 kb in MA and 324 kb in CS (Table S2). Although some regions remained without coverage, 7.2% and 9.1% of the genome of MA and CS, respectively, the achieved density was suitable for candidate gene identification.

### 3.3. QTL Analysis

In this analysis, we reported significant as well as suggestive QTLs regardless of their phenotypic effect or reproducibility because, in other progenies or different genetic backgrounds, the suggestive or barely reproducible QTLs here reported might exist with different effects over the phenotype, with different reliability and reproducibility values. Six, ten, and eight QTLs were identified for SEDW, SEDN, and SEDA, respectively, and four, five, and five of them were stable, respectively (Table 1).

**Figure 3:**
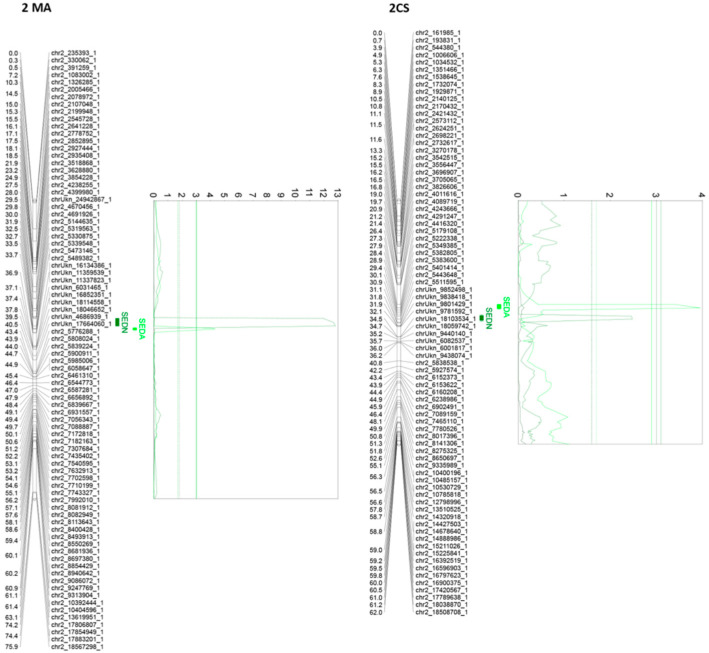
Principal QTLs detected in chromosome 2 for seedlessness subtraits SEDN and SEDA in the MA × CS experimental population. SEDN: number of seeds per berry; SEDA: average fresh weight of one seed. 1-LOD and 2-LOD support confidence intervals were drawn. Thresholds for suggestive (dashed lines) or significant (continues lines) QTLs were established by a permutation test with 10,000 iterations. Both parental maps were represented. MA: Muscat of Alexandria; CS: Crimson Seedless.

**Figure 4:**
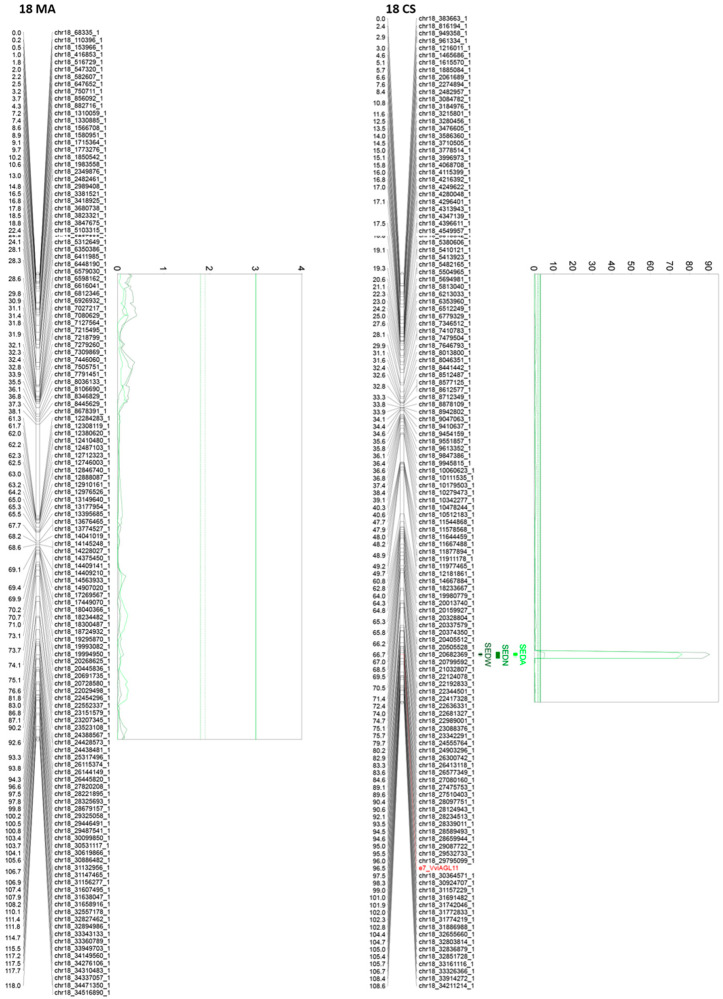
Principal QTLs detected in chromosome 18 for seedlessness subtraits SEDW, SEDN, and SEDA in the MA × CS experimental population. SEDW: seed fresh weight per berry; SEDN: number of seeds per berry; SEDA: average fresh weight of one seed. 1-LOD and 2-LOD support confidence intervals were drawn. Thresholds for suggestive (dashed lines) or significant (continues lines) QTLs were established by a permutation test with 10,000 iterations. Both parental maps were represented. MA: Muscat of Alexandria; CS: Crimson Seedless.

Of all the 24 identified QTLs for the seed phenotypic subtraits, 16 are unique, whereas the rest of the QTLs are for more than one trait. Interestingly, QTLs identified on chromosome 2, in both parental genotypes, are very close or adjacent QTLs that probably define a unique region associated with both SEDN and SEDA. However, their effects are different. The same QTL for SEDN explains 9.2 and 1.6% of the phenotypic variability in MA and CS, respectively. Two additional regions in chromosome 13 of MA contain co-positioning QTLs for SEDN and SEDW at 1.3 cM and for SEDA and SEDW at 61 cM. One region in chromosome 14 of CS contains co-positioning QTLs for SEDA and SEDW, and one region in chromosome 18 of CS contains co-positioning QTLs for SEDW, SEDN, and SEDA (Table 1 and Figure 3, Figure 4 and Figure S5). 

The most relevant QTL for SEDW and SEDA corresponds to the *SDI locus* [10], today known as the *VviAGL11* [9] gene, which is present in an extreme of chromosome 18 and segregates from CS, explaining 48.4%, 42.5%, and 3.5% of the total phenotypic variation for SEDW, SEDA, and SEDN respectively (Table 1, Figure 3, Figure 4 and Figure S5). The second most interesting QTL, in terms of its effect and reproducibility, was located in chromosome 2 and segregating from MA. This QTL explains 9.2% and 3.2% of the total variability of SEDN and SEDA, respectively (Table 1, Figure 3, Figure 4 and Figure S5). All the other identified QTLs individually explain less than 3% of the total phenotypic variation, which is not encouraging for assisted selection purposes; however, most of them have a stable effect over the phenotype (Table 1). Overall, QTL results showed that the genetic architecture of seedlessness is complex, regardless of the existence of a major gene that contributes to almost half of the total variation for SEDW and SEDA. Additional loci were identified and characterized in terms of their effect, reliability, and stability.

### 3.4. Multiloci Assisted Selection

Considering the additive effect of QTLs, the developed predictive model includes the simultaneous evaluation of multiple QTLs, which were selected methodically and added one by one according to their phenotypic individual effect (Table 1) in decreasing order (Table 2). With each added marker, the size of the population that represents the ideotype is divided by half; thus, due to the size of the population (*n* = 573), the effect of more than five markers was difficult to test by one-way ANOVA. Starting with the largest-effect QTL for each trait that explains 52.11%, 9.15%, and 43.87% for SEDW, SEDN, and SEDA, respectively, and with five markers for each trait, we were able to increase the total phenotypic variation up to 55.98%, 21.72%, and 51.92% for these traits (Table 2). In phenotypic values, SEDW decreases 25.2% compared to the use of only the *VviAGL11*-derived marker, and for SEDN and SEDA, the phenotypic values decrease 32.4% and 36.2% compared to the sole use of the largest-effect QTL.

### 3.5. QTL Confidence Intervals and the Identification of Candidate Genes

For SEDW, the average size of confidence intervals is 914 kb, with minimum and maximum sizes of 101 and 2438 kb. In total, confidence intervals for SEDW result in 5484 kb, which contains 384 genes according to the latest genome annotation (Vcost.V3, [33]). For SEDN, the average size of confidence intervals is 789 kb, with minimum and maximum sizes of 128 and 3211 kb. In total, the confidence intervals for SEDN are 7892 kb in size, corresponding to 851 genes. For SEDA, the average size of confidence intervals is 1461 kb, with minimum and maximum sizes of 195 and 4201 kb. In total, the confidence intervals for SEDN are 11,690 kb in size, corresponding to 1105 genes (Supplementary Table S3).

For SEDW, SEDN, and SEDA, the number of candidate genes, based on their functional annotation and category, is 20, 43, and 50, respectively (Supplementary Table S4), 74 of which are unique due to the co-positioning of several QTLs. In the large-effect QTL of chromosome 18 for SEDW and SEDA, the *VviAGL11* (annotations Vitvi18g02133 and VIT_18s0041g01880 in Vcost.V3 and V2, respectively) gene is still the only candidate gene.

For the second most important QTL, SEDN in chromosome 2, the most important candidate gene is a *YABBY* transcription factor (annotations Vitvi02g00510 and VIT_02s0154g00070 in Vcost.V3 and V2, respectively) involved in the abaxial cell fate determination during embryogenesis and organogenesis. *YABBY* regulates the initiation of the embryonic shoot apical meristem (SAM) development [40,41] and is required during flower formation, *INNER NO OUTER* (*INO*) is a *YABBY* protein that is essential for the initiation and development of the outer integument of ovules [42].

Several other candidate genes are related to development, principally transcription factors such as *WUS*, *bHLH*, *MADS-box*, *OVATE*, *ABA insensitive*, *FRIGIDA*, *MYB*, and *ZIP*. Several other candidate genes are involved in growth regulation or hormone signaling, such as through gibberellin, brassinosteroid receptors, or auxin signaling. Genes involved in the regulation, synthesis, or transport of flavonoids and anthocyanins such as those in the *MYB* family were also identified (Supplementary Table S4).

### 3.6. Development of Markers for Assisted Selection

#### 3.6.1. Microsatellite 5U_VviAGL11 

**Figure 5:**
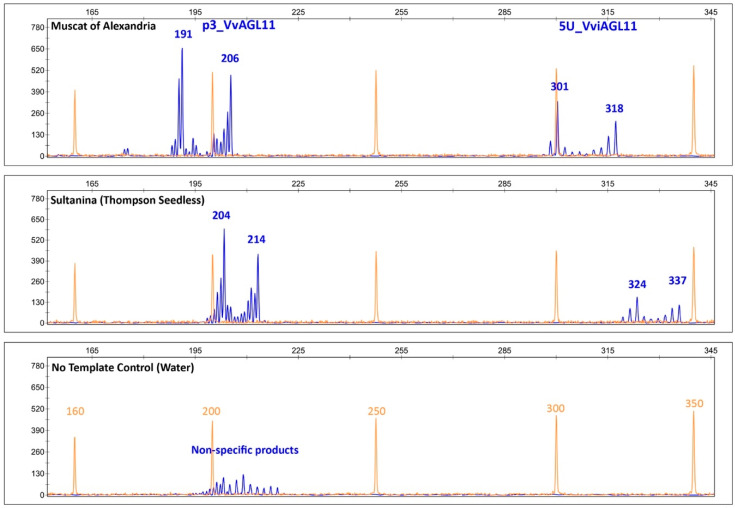
Capillary electrophoretic profile of SSRs p3_VvAGL11 and 5U_VviAGL11. Orange: the profile of the GeneScan 500 LIZ Size Standard (Applied Biosystems, Foster City, CA, USA). Blue: the specific profile of p3_VvAGL11 and 5U_VviAGL11 markers that amplify, respectively, 191/206 bp and 301/318 bp alleles in Muscat of Alexandria and 204/214 bp and 324/337 bp in Sultanina. No template control unspecific product amplified by p3_VvAGL11 is shown. Amplified products contain the M13 tail (+18 bp) labeled with FAM fluorescent dye.

The developed marker produces specific PCR products that revealed a diverse range of sizes between 285 and 343 bp (267 and 325 bp without the 18 bp of the universal M13 tail). This marker is perfectly suitable for use with or without the universal M13 tail, although we present results obtained with the tail. The developed marker contains the previously described marker, p3_VvAGL11, and includes a third (GA)n repeat tract that also revealed variations when we sequenced the *VviAGL11* 5′UTR region (Supplementary Figure S1). Amplicons are sharp and with significantly less stuttering, which reduces the effort to identify the correct pattern and allele calling, especially with low quality/quantity templates. An additional feature of 5U_VviAGL11 is that no template controls (negative controls) do not produce non-specific products that are produced in higher frequency by p3_VvAGL11 (Figure 5). Together, sharpness and specificity reduce errors in automatic allele calling and eases post-electrophoretic analysis.

**Figure 6:**
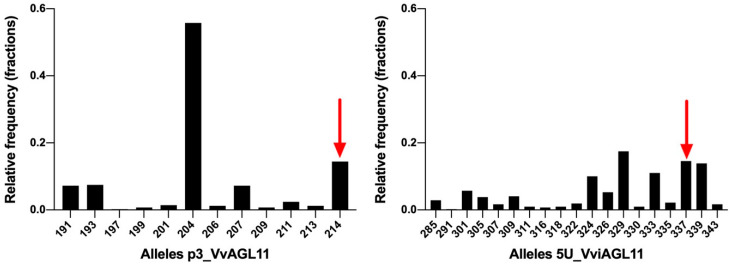
SSRs p3_VvAGL11 and 5U_VviAGL11 allele distribution in a collection of 209 unique *Vitis vinifera* individuals. Alleles include the M13 tail (+18 bp). Bars indicated by a red arrow correspond to the unique seedless allele.

In a *Vitis vinifera* background, results showed that SSR 5U_VviAGL11 is more informative than p3_VviAGL11. It produces seven more alleles, 19 in total (Figure 6). Considering other *Vitis* species, p3_VvAGL11 reveals two additional alleles, whereas 5U_VviAGL11 reveals five additional alleles (Table S5). In other members of the Vitaceae family the SSR 5U_VviAGL11 did not produced amplicons (Table S5). Considering only a *Vitis vinifera* background, the Polymorphism Information Content was 0.627 for p3_VvAGL11 and 0.886 for 5U_VviAGL11 (Figure 6 and Supplementary Table S6). Both markers identify the seedless allele of 214 and 337 bp in p3_VvAGL11 and 5U_VviAGL11, respectively (196 and 319 without the M13 universal tail) (Figure 5 and Figure 6).

#### 3.6.2. SNP e7_VviAGL11 to Identify the Arg-197-Leu Mutation [G/T] in VviAGL11 by qPCR-HRM

**Figure 7:**
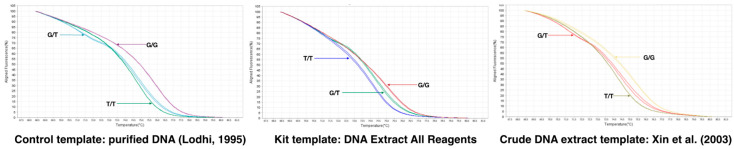
qPCR-High Resolution Melting analysis for the detection of the SNP [G/T] with the e7_VviAGL11 marker. Comparison of the aligned melt curves between homozygous seedless (RU × SU:16.48 and 33.34 experimental genotypes), heterozygous seedless (Sultanina and Crimson Seedless), and seeded genotypes (Muscat of Alexandria and Red Globe). The developed marker is suitable for applications in high-throughput routines sustained by qPCR-HRM and commercial (DNA Extract All Reagents from Applied Biosystems, Foster City, CA, USA) or lab-made crude DNA extracts (protocol described by Xin et al. [27] as a template.

Since the Arg-197-Leu mutation is causal with respect to the seedless phenotype, we developed a quantitative PCR (qPCR) high-resolution melting (HRM) curve analysis (qPCR-HRM) methodology that combines qPCR and HRM to obtain a rapid and cost-effective method suitable for testing a large series of samples. Homozygous seeded [G/G] and seedless [T/T] genotypes produce amplicons with melting temperatures (Tm) of 76.0 and 74.7 °C, respectively, whereas the heteroduplex presented a specific curve with two melting peaks of 74.7 and 71.9 °C (Figure S6). The developed marker is suitable for the use of a gDNA template purified by commercial kits, including the popular [26] CTAB-based extraction protocol, the crude extract-based protocol available in commercial kits, and the protocol described by Xin et al. [27], with minor modifications described in the Materials and Method section. The three variants were easily and unequivocally distinguished (Figure 7 and Supplementary Figure S6). 

#### 3.6.3. SNP e7_VviAGL11 to Identify the Arg-197-Leu Mutation [G/T] in VviAGL11 by Tetra-Primer ARMS-PCR

**Figure 8:**
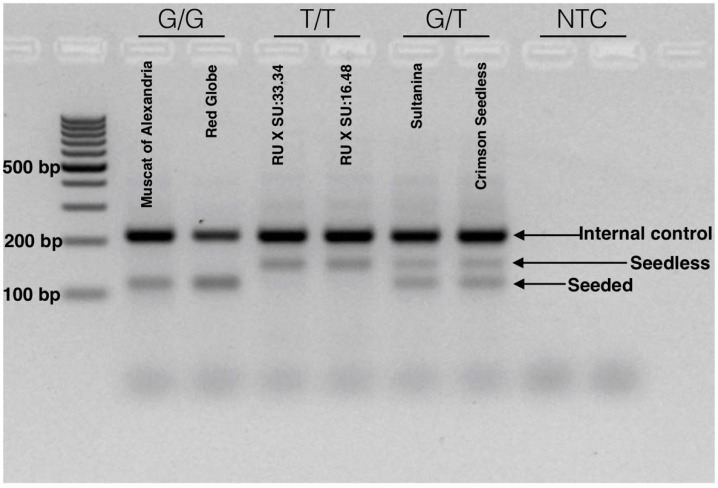
Tetra-ARMS PCR electrophoretic analysis for the detection of the SNP [G/T] with the e7_VviAGL11 marker. The SNP marker is responsible for the Arg-197-Leu mutation in the coding sequence of VviAGL11. The electrophoretic profile compares seeded genotypes (Muscat of Alexandria and Red Globe) with homozygous seedless (RU × SU:16.48 and 33.34 experimental genotypes) and heterozygous seedless (Sultanina and Crimson Seedless) genotypes. Seeded and seedless alleles produce specific products of 115 and 150 bp, respectively. Both alleles amplify the internal control product of 212 bp. A developed marker is suitable for the application in simple routines based on standard PCR and agarose electrophoresis.

After optimization and validation, we defined a simple method for the identification of the Arg-197-Leu mutation in *VviAGL11* that is causal with respect to the seedless phenotype. The reaction is run in a single tube, in a single PCR step, and genotype variation can be visualized directly using common agarose gel electrophoresis. The reaction produces specific 115 and 150 bp alleles for the homozygous [G/G] seeded and [T/T] seedless genotypes, respectively. Heterozygous genotypes [G/T] produce both specific products (Figure 8). The reproducibility of the methodology was validated in a panel of 90 MA × CS segregants. The simplicity of the method enables small breeding programs to apply assisted selection without either the habilitation of high cost equipment, such as a qPCR thermal cycler or a fragment analyzer, or highly trained personnel.

### 3.7. Evaluation of the Performance of SSR 5U_VviAGL11 and SNP e7_VviAGL11 for the Prediction of the Seedless Phenotype in the Context of Breeding

Both SSRs, p3_VvAGL11 and 5U_VviAGL11, deliver exactly the same information in regard to the seedless allele, 214 bp for p3_VvAGL11 (196 bp without the M13 tail) or 337 bp for 5U_VviAGL11 (319 without the M13 tail) (Figure 5 and Figure 6, Table 3, and Supplementary Table S5). Due to the dominance effect of the seedless allele, both homozygotes and heterozygotes containing the seedless allele can be considered seedless. Thus, the genotype can be analyzed as a binary variable: with the seedless allele or without the seedless allele. At the phenotype level, seedlessness is a quantitative trait; however, due to the partial dominance of the seedless allele and the bimodal distribution of most of the seedless subtraits (SEDW and SEDA, Figure 1 and Supplementary Figure S3), the phenotype can be classified as a binary variable with an arbitrarily established cutoff value (0.045 g in this work): containing seed rudimental traces or containing completely developed seeds. Thus, the evaluation of markers for seedlessness assisted selection was treated binarily, and its evaluation was treated in consequence in 2 × 2 contingency tables. For a collection of 209 varieties, 5U_VviAGL11 presented a sensitivity of 1.0, a specificity of 0.938, and an accuracy of 0.953. A positive predictive value of 0.836 and a negative predictive value of 1.0 means that some individuals predicted as seedless present completely developed seeds (false positives) (Supplementary Table S7). This rate of false positives drops to zero with the use of SNP marker e7_VviAGL11 (Table S7). These results reflect that false positive individuals contain the seedless 5′UTR region linked to the seeded coding sequence. In this set of varieties, the SNP marker e7_VviAGL11 showed perfect prediction values, which is expected for a causal SNP.

Material includes a collection of 209 varieties, 48 segregants from a Red Globe (RG) × Autumn Royal (AR) cross, 48 segregants from a Red globe (RG) × Regal Seedless (RS) cross, breeding material composed of 174 segregants issued from 55 different crosses, 9 other *Vitis* species and 4 other Vitaceae members, that were genotyped by p3_VvAGL11 and 5U_VviAGL11 SSRs as well as by the e7_VviAGL11 SNP [G/T]. NA: no amplification.

**Figure 9:**
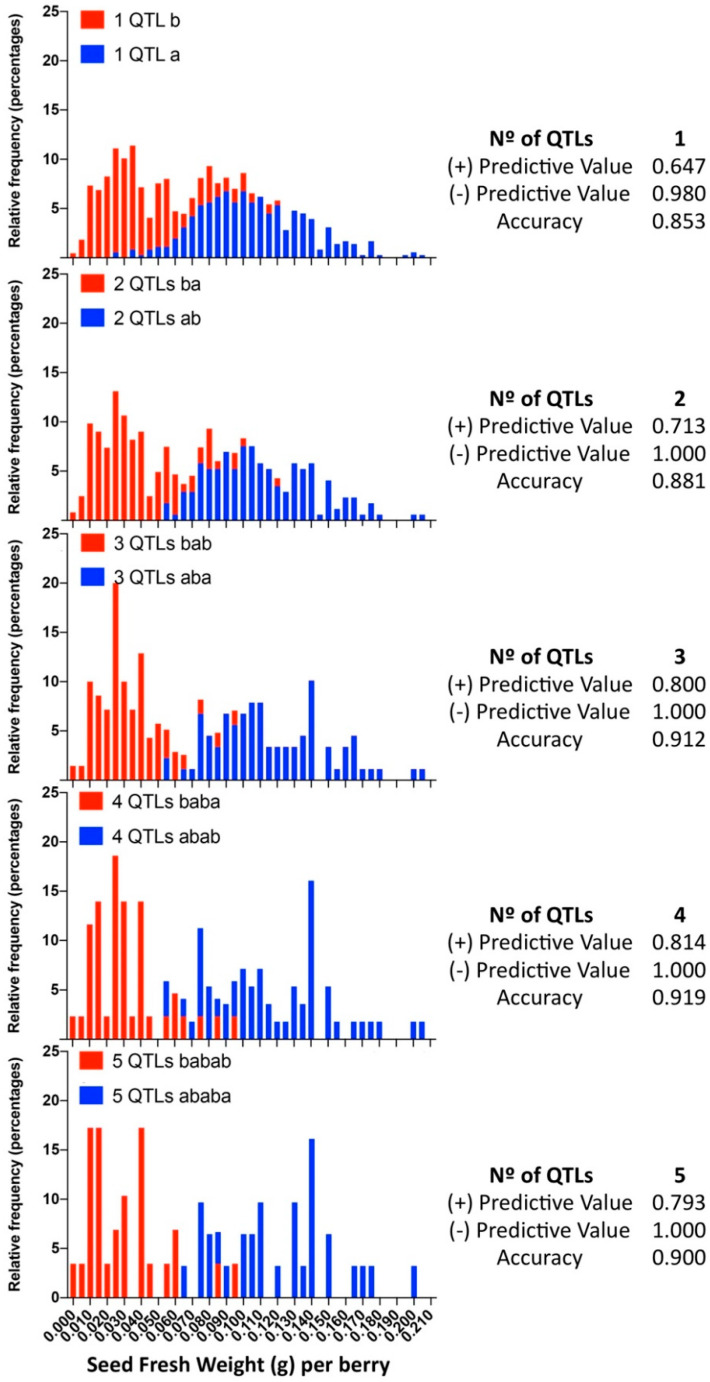
Frequency distribution of the MA × CS subpopulation sets selected by the most favorable alleles combining one to five QTLs. For each QTL, the most favorable allele combination is defined in Table 2, and the relative frequency of individuals is shown in red bars. Blue: the individuals selected by the opposite allele combination. The positive and negative predictive values were calculated from contingency tables.

When we evaluated the prediction performance in small progenies of different genetic background, RG [324/329 defined by 5U_VviAGL11] × AR [337/339] and RG [324/329] × RS [329/337], minor differences were identified between the SSR 5U_VviAGL11 and the SNP e7_VviAGL11, which correspond to individuals that present a recombination between the 5′UTR region and exon 7 of the coding sequence. Compared to the results obtained with 209 varieties, the most noticeable effect is the variable false positive and negative rates for individuals that were predicted as seedless but contain rudiments slightly over the cutoff value (0.045) and individuals that were predicted as seeded but contain SEDW values below the cutoff value (Tables S5 and S8–S10). When we evaluated the prediction performance in the largest progeny, MA [301/318] × CS [329/337], which was evaluated only with SNP e7_VviAGL11, the evaluated sensitivity value was 0.953, the specificity was 0.819, and the false positive and negative rates were 18.12% and 4.73%, respectively (Tables S5 and S11), although these values might be adjusted as the cutoff value is moved, which reflects the classification problem of when both classes are overlapping and, in this sense, reflects the true nature of this complex trait. Genotyping of the complete MA × CS population with e7_VviAGL11 and the respective phenotypic distribution of both genotypic classes show the overlapping classes (Figure 9). The application of the multiloci or multi-QTL selection model, increasing selection levels by adding QTLs one by one, the overlapping effect tends to disappear, as the positive and negative predictive values tend to increase as more markers are included in the model. These results confirm the limitations of the *VvAGL11* gene as the sole tool for assisted selection and confirms the complex nature of the seedless subtraits.

## 4. Discussions

### 4.1. Trait Variation

Spearman’s correlation (Figure S4), as well as phenotypic distribution (Figure 1 and Figure S3), displayed differences among seasons for SEDW, SEDN, and SEDA. However, it was more noticeable in SEDN, which showed the lowest correlation value (0.3484) in comparison to SEDW (0.653) and SEDA (0.6183). Complementarily, a combined analysis of variance for SEDW, SEDN, and SEDA calculated for the segregant individuals and seasons indicated a significant difference (*p* < 0.0001) in terms of genetic variation. Nonetheless, it manifested the complexity of these seed phenotypic subtraits due to the considerable interaction between genotypes and seasons as much as experimental error. Thus, these analyses reflect that the environmental effect is relevant for the expression of these seed phenotypic subtraits. Seed development is the result of ovule fertilization that depends on pollen tube elongation. Immediatly after pollintation, the pollen grain absorbs moisture from the stigma and forms the pollen tube, this process has its optimum between 25 and 30 °C, temperatures below 10 °C or above 35 °C inhibit pollen germination [43]. In this work, seasons 2015 and 2018 were characterized by air temperatures below or above optimum during several days at flowering period (between 5 and 18 November, Supplementary Figure S7). Ebadi et al. ([44]) reported that cold temperatures a couple of days before flowering results in a significant decrease in pollen germination and pollen tube growth and, Ewart et al. ([45]) showed that lower temperatures during the period from one week before bloom until *véraison* resulted in half of the number of seeds per berry compared to bloom at optimum temperatures. Overall, this information is useful to understand why some seedless varieties, which do not have a stable phenotype between seasons, vary between small rudiments to even palatable rudiments that are generally eliminated by applying gibberellic acid [46].

### 4.2. The Complex Genetic Architecture of Seedlessness

Stenospermocarpic seedlessness is defined at phenotypic level by several aspects or sub-traits, originally by Stout [8], and in this context by our data it appears that it is not a monogenic or quasi-monogenic trait, as proposed earlier [9], in relation to the major role of the *VviAGL11* gene. The QTL analysis reveals that seedlessness is a quantitative complex trait. Up to 15 unique QTLs, 11 of which are stable, were identified for seedless subtraits (SEDW, SEDN, and SEDA) (Table 1). Compared to previous works based on QTL mapping experiments for complex traits in grapevine [47,48,49], such as seedless subtraits, results attract attention by the number of detected QTLs but also by their very small phenotypic effect, with the exception of the *VviAGL11* locus that controls SEDW and SEDA, explaining 48.4% and 42.5% of the total variation for these traits, respectively (Table 1). We reported all QTLs regardless of their significance level (suggestive or significant according to [31]). Suggestive QTLs reported in this work can have a different impact on other genetic backgrounds, and this information might be important for the grapevine community working on genetics and breeding in order to identify the most favorable alleles for breeding.

We reported the stability of the identified QTLs and found that 4/6, 5/10, and 5/8 QTLs for SEDW, SEDN, and SEDA, respectively, were identified as stable based on the reproducibility of results over 2–4 seasons. Of the QTLs, one in six, four in ten, and two in ten for SEDW, SEDN, and SEDA, respectively, were identified as not reproducible between seasons (but with support from BLUPs and a single independent season). Finally, one in six, one in ten, and one in eight QTLs for SEDW, SEDN, and SEDA, respectively, were identified as supported only by BLUP analysis (Table 1). Considering the size of the population (*n* = 573) and the density of mapped markers (~1 cM/SNPs), QTLs detected only in one season or only with BLUPs might truly exist as unstable QTLs rather than being fake associations.

In detail, the segment of chromosome 2, between 4.4 and 5.5 Mbp (Supplementary Table S4), contains QTLs that explain 1.6% of the variation in CS and 9.2% of that in MA for SEDN, and 3.2% for SEDA in MA was previously described by Costantini et al. [49], who also identified a significant QTL for SEDN and a flowering time that explains ~20% of the variation in a Italia × Big Perlon progeny. The region was also studied by Doligèz et al. [50], who identified QTLs for SEDW and SEDN that explain 39.6% and 37.1% of the variation, respectively, in a Syrah/Grenache progeny. Massonet et al. [51] reported recently that a shorter fragment of the same region, between 4.8 and 5.1 Mbp, contains genes and polymorphisms related to sex determinism in grapevine. As sex-related genes (fertility and sterility genes) directly affect the number of seeds, female flowers need the external pollination and therefore have a less effective fertilization. In the MA × CS population, the QTL for SEDN located in this region was stable in three of the four evaluated seasons, which explains, in part, the poor correlation between seasons for this trait (Supplementary Figure S4). The difference in the proportion of explained variance from these cited works might be explained by the Beavis effect [52]. Small progenies greatly overrate estimated effects (163 in [49] and 96 in [50]). The group of Doligèz et al. [47] also identified two other QTLs that were confirmed by this study with much higher resolution. A QTL was detected for SEDN in chromosome 1, between 4.5 and 4.9 Mbp (Table S4), that explains 2.8% of the variation, and a QTL for SEDW in a segment between 3.8 and 7.6 Mbp explained ~13% of the total variation in a Syrah × Grenache progeny. The QTL for SEDW that explains 0.9% of the variation was detected in chromosome 4 between 19.6 and 20 Mbp (Suppplementary Table S4), and QTLs for SEDN and SEDW in a segment between 18 and 20 Mbp explained 23.9% and 27.3% of the variation in a Syrah/Grenache progeny. The rest of the described unique QTLs (12) in this work are new and have not been previously reported.

The most important difference in this work, in relation to previous analyses, is the size of the experimental population that enabled the construction of robust and reliable parental maps with a density suitable for candidate gene identification. Most of the previous works were based on populations of less than 200 individuals (139 in [50], 118 in [48], 163 in [49], 139 in [9], and 96, 139, and 174 in [50]); here, we used a population of almost 600 individuals, which allowed us to identify QTLs with a lesser but stable effect (not severely overrated). The same QTL analysis based on 300 individuals randomly selected from the MA × CS progeny allowed for the identification only of major-effect QTLs. With 150 individuals, we only detected the VviAGL11 loci for SEDW, but we also detected spurious associations.

### 4.3. Candidate Genes

The identification of candidate genes for seedlessness has three main goals: first, the identification of putative causal genes results in a biological predictive model rather than simply an association model; second, it reduces the chance of recombination between adjacent markers and the causal gene, making predictability more specific and accurate when intragenic markers are designed; and third, knowledge of the causal gene delivers a working hypothesis for more advanced breeding strategies such as genome editing.

Considering the nature of seed development and seedlessness aspects based on the seed number and seed size, but also in the seed lignification degree and embryo abortion and development, several aspects of the seed physiology and flower development processes were considered: from hormones metabolism, transport, signaling, and homeostasis, embryo development, floral initiation, floral organ development, and seed coat and endosperm development to anthocyanin synthesis, transport, and accumulation. Taking this into consideration, in the present study, we identified 75 unique candidate genes for SEDW, SEDN, and SEDA (Supplementary Table S4).

In this work, the *VviAGL11* gene was identified as the sole candidate gene from the major QTL identified in chromosome 18 for SEDW and SEDA, and this locus has previously been described [9,49] and later validated at the functional level in grapes [18,53] and in a heterologous system [17]. More recently, Royo et al. [16] pointed out an SNP mutation in exon 7 as the factor responsible for the phenotype. Together, these results show that QTLs are accurately indicating causal genes [54]. For SEDN, *VviAGL11* has a minor role (3.5%, Table 1), compared to reports [48,49,50] showing that the *locus SDI* (now known as *VviAGL11*) explained up to 12%, 40.8%, and 59% of the respective phenotypic variance for SEDN. This difference might be due to the use of SEDW as a covariate for the MQM analysis of seed number and seed size in this study, but also by the overestimated effects due to the size of the progenies used in these studies. 

For SEDN, the QTL analysis shows that the largest contribution comes from chromosome 2 of Muscat of Alexandria, explaining up to 9.2% of the total variation. Practically the same QTL was identified in Crimson Seedless, but only explaining 1.6% of the variation for SEDA (Table 1). Considering the reference genome (Pinot Noir PN40024 [25], in such a confidence interval, the most important candidate genes for SEDN and SEDA are a transcription factor from the *YABBY* family and a β-vacuolar processing enzyme. *YABBY* is involved in the abaxial cell fate determination during embryogenesis, and it is required during flower formation and development for the patterning of floral organs [55]. *YABBY* has several interactions with members of the ABC model of flower development. It is a positive regulator of *AP3* and *PI* (class B genes [55]), *AP2*, and *LUG* (class A genes [41]), which in turn can repress *AG* (class C [56]). Considering these interactions with members of the ABC floral development model, an interaction of this *YABBY* with *VviAGL11* (of class D) would not be surprising and, in that sense, its action would be upstream of *VviAGL11*. In the grapevine, Costantini et al. [49] identified a QTL for seed number and flowering time very close to VVIB23 locus. Later, Batillana et al. [57] indicated that a VVIB23 SSR marker, contained in the 3′UTR of the *YABBY* gene, has specific variations associated with specific phenotypes of the flower sex locus. More recently, Zhang et al. [58] showed that *VvYABBY3* (from the same VVI23 locus in chromosome 2) has an increased expression at the beginning of ovule abortion and Massonnet et al. [51] found that *YABBY* was more highly expressed in male flowers compared to female and hermaphrodite flowers at pre-blooming and anthesis stages, suggesting that *YABBY* is associated with female sterility in grape, inhibiting ovule development. Transgenic expression of VvYABBY4 (that has a similar expression pattern as *VvYABBY3*) in tomatoes conferred reduced seed sizes associated with smaller endosperm cells, but without affecting the number of seeds per fruit [58]). In *Vitis*, *VvβVPE,* a vacuolar processing enzyme not located in chromosome 2, was described as having a caspase-1-like activity with the ability to regulate programmed cell death (PCD) and putatively playing an essential role in the development of stenospermocarpic seedless grapes ovules [59]. Tissue-specific expression analysis of three *Vitis vinifera VPE* genes (*VvβVPE*, *VvγVPE*, and *VvδVPE*) showed that *VvδVPE* was only expressed in flowers, buds, and ovules. *VvγVPE* was expressed in various tissues, and *VvβVPE* was expressed in roots, flowers, buds, and ovules [59]. In addition, transcription analysis showed that *VvβVPE* expression in seeded grapes increased significantly 30 days after full bloom (DAF), close to the timing of endosperm abortion at 32 DAF, while it was weakly expressed in seedless grapes [59]. These results were validated [60,61], suggesting that *VvβVPE* is related to ovule abortion in stenospermocarpic seedless grapes. In *Arabidopsis*, a *δVPE* was identified as essential for seed coat formation at early stages of seed development [62]. Interestingly, as mentioned in the preceding section, a recent work from Massonet et al. [51] proposed several new genes and polymorphisms associated to the sex determinism in grapevines. Among the most interesting and new proposed genes, *INAPERTURATE POLLEN 1* (*INP1*) and *adenine phosphoribosyltransferase 3* (*APT3*) were discovered and annotated only in the Cabernet Sauvignon genome sequenced and assembled by single molecule real-time sequencing, haplotype phasing and transcriptome sequencing based in long reads [63,64].

Massonnet et al. ([51]) presented a model that explains the sex determination in grapevine that involves putatively the combined action of several genes mapped within the 4.8 and 5.1 Mbp region of chromosome 2. Among the best candidate genes involved in this model that explains male or female sterility are *INP1*, *APT3* and *YABBY* that was discussed above. Pollen grains are surrounded by a very resistant wall interrupted by apertures, areas where the wall is thinner or softer, that play a key role because the pollen tube growth is initiated at these apertures, through them the pollen tube can reach out from the inside and transport the sperm cells to the female structures [65]. Pollen aperture factor *INP1* is absolutely required for aperture formation [66]. Results of Massonnet et al. [51] show that an 8 bp deletion in *INP1* in homozygous state causes male sterility, to compensate the non-functional protein the female flowers express *INP1* to higher levels compared to male or hermaphrodite flowers, resulting in a less effective fertilization and a reduced number of seeds. *Adenine phosphoribosyltransferase 3* catalyzes a reaction that inactivates cytokinins by phosphoribosylation, loss of activity leads to excess accumulation of cytokinin, evoking myriad cytokinin-regulated responses [67]. Cytokinins positively regulate ovule formation, mutants with a reduced capacity of cytokinin production or perception exhibit a dramatic reduction in ovule number [68]. Massonnet et al. [51] showed that *APT3* is highly expressed in male flowers that probably results in cytokinin inactivation affecting the number of ovules per pistil and therefore the number of seeds.

From minor-effect QTLs, we identified several candidate genes, among them members of the MYB family. The TT2 gene, an R2R3 MYB, is a key regulator of the accumulation of proanthocyanidins in developing seeds [69]. The R2R3 MYB transcription factor regulates seed size in Arabidopsis thaliana [70], MYB89 represses seed oil accumulation [71], MYB7 is involved in the regulation of flavonol biosynthesis and plays a role in ABA signaling, blocking the expression of the bZIP transcription factor ABI5 [72], Myb domain protein 3R-5 regulates plant organ growth affecting G2/M-specific genes, and mutants of the three repressor-type *MYB3R* genes displayed enlarged leaves, embryos, and seeds [73]. *MYB1*, an *R2R3-MYB* type, regulates anthocyanin biosynthesis [74] and *MYB 77*, an R2R3 member, modulates auxin signal transduction [75]. At the same time it has been reported that MYB and basic helix–loop–helix (bHLH) transcription factors regulate *TTG2* expression [76]. *TTG2* is involved in flavonoids synthesis which, in turn, regulates communication between seed coat and endosperm, controlling seed size [77].

A *TRANSPARENT TESTA12* gene was also identified as a candidate gene in chromosome 12 of Crimson Seedless, and in *Arabidopsis*, it is expressed in ovules and developing seeds and encodes a transporter for flavonoid sequestration in vacuoles of the seed coat endothelium, interfering with seed dormancy [78].

Additionally, a couple of *bHLH* candidate genes were identified, one for SEDW and SEDN in chromosome 13 of Muscat of Alexandria and another for SEDW and SEDA in chromosome 14 of Crimson Seedless. The bHLH transcription factor family is important for seed development. *RETARDED GROWTH OF EMBRYO1* is expressed in endosperm to control embryo growth [79] in *Arabidopsis*, and in grapevine embryos, the overexpression of grape *bHLH VvCEB1* affected embryo development and increased cell size [80]. *VvCEB1* is a *bHLH* member that increased number and seed yield in *Arabidopsis* [81].

Another group of transcription factor identified within QTLs is the *ZIP* family, two for SEDW and SEDA in chromosome 13 of Muscat of Alexandria and one for SEDN and SEDA in chromosome 8 of Muscat of Alexandria and Crimson Seedless, respectively. During the maturation phase of seed development in *Arabidopsis*, bZIP53, bZIP10, bZIP25, and ABI5 transcription factors participate in regulating this process [82,83]. They lead to the activation of seed storage protein genes [84] that accumulate in the endosperm. The second family of transcription factors that are essential for the correct completion of seed maturation is the ABA-insensitive. ABI3 increases the expression of maturation genes [85], and ABI4 regulates primary seed dormancy [86].

Two GRAS members were identified as candidate genes. *SCL6* in chromosome 2 of Muscat of Alexandria and Crimson Seedless for SEDA and in chromosome 2 of Muscat of Alexandria for SEDN. *SCL29* in chromosome 9 of Muscat of Alexandria and *SCL9* in chromosome 8 of Crimson Seedless for SEDA. Playing crucial roles in plant growth and development, scarecrow-like protein 29 ranges from GA signaling, root radial pattering, light signal transduction, and axillary shoot meristem formation [87].

Another interesting candidate gene for SEDN was found in chromosome 1 of Muscat of Alexandria from *the Wuschel-related Homeobox* (*WOX*) family, a class of transcription factors involved in the early stages of embryogenesis [88] and floral organ pattering [89]. *PRETTY FEW SEEDS2*, from the *WUS* family gene, encodes an *Arabidopsis* homeodomain protein that regulates ovule development, altering *AGAMOUS* expression [89,90]. In rice, overexpression of *WOX3* represses *YAB3* (*YABBY* gene), which is required for leaf development [91]. In the same way, it is possible that *WUS* in chromosome 1 might be in control of *YABBY* in chromosome 2.

The AINTEGUMENTA transcription factor was also identified in chromosome 9 of Muscat of Alexandria for SEDA. It is involved in ovule development and in the initiation and growth of floral organs, acting as a negative regulator of *AGAMOUS* [92]. It is also required for integument initiation in ovules [93]. The integument transforms into a seed coat, so *AINTEGUMENTA* could be part of a molecular mechanism controlling grape seed size.

Finally, we also identified *TM6 APETALA 3* as a candidate gene for SEDW in chromosome 4 of Crimson Seedless. This transcription factor is a member of the MADS-box family that has been shown to play key roles in the specification of petal and stamen identities [94,95]. De Martino et al. [96] describe the loss-of-function phenotypes for *TM6* and *TAP3*, which indicates that these tomato genes play distinct roles in flower development. *TAP3* is required to specify both petal and stamen identity, whereas *TM6* appears to play a role predominantly in stamen. The stamen is the reproductive organ that produces pollen, and the seed number and development depends on the effective pollination of ovules [97]. In this way, the determination of the stamens could indirectly determine the formation of seeds.

### 4.4. Multi-QTL Assisted Selection

In a sufficiently large progeny such as the MA × CS (*n* = 573), applying the *VviAGL11* gene for the segregation of seeded from seedless individuals, the first group has an SEDW of 0.1052 ± 0.0320 (g) on average, while the second group has an SEDW of 0.0410 ± 0.0256 (g) (Table 1). However, the phenotypic distribution of both groups clearly shows overlapping classes (Figure 9) that result in up to 18.1% and 4.7% of false positive and negative rates, respectively, comparing the predicted phenotype with the arbitrary cutoff value of 0.045 g (Supplementary Table S11). In the same way, in small progenies, the rate of false positives or negative varies between 3% and 24% (Supplementary Tables S8–S10), reflecting that the major effect of *VviAGL11* is not accurate enough by itself for the selection of F1 offspring.

Considering the discovery of 15 unique QTLs for seedless subtraits, most of them are of very low effect but stable. As a proof of concept, we tested a model that benefits from the additivity of QTLs that are independently inherited (i.e., not linked). We succeeded in improving the accuracy of the positive predictive value, from 0.647 with the *VvAGL11*-based marker alone up to 0.814 with three additional markers (Figure 9).

With the selection of individuals that contain the most favorable allele combination for up to five markers, we were able to select less than 5% of the population for SEDW, SEDN, and SEDA, respectively (Table 2). Hypothetically, this represents up to 500 individuals for programs that generate 10,000 seedlings every year. Selecting for a second trait with five additional markers can result in 15 individuals to be established in the field for further evaluations. These 15 individuals can be propagated earlier for field evaluations, reducing the timing of breeding cycle [98].

### 4.5. Development of Robust, Simple, and Accurate Markers for Assisted Selection of Seedlessness

Assisted selection of seedlessness is currently applied based on the use of SSRs p3_VvAGL11 ([9,11,99,100,101]). In general, the use of this marker enables the selection of the best offspring candidates at early stages of the breeding pipeline: in vitro, when seedlings come from seedless × seedless crosses, or at early stages of development (with two or three expanded leaves), when seedlings come from seed germination. In crosses derived from seedless and seeded, the trait segregates 1:1 due to the partial dominance of the seedlessness allele, enabling a reduction of up to 50% in offspring. In crosses derived from seedless × seedless, seedlessness segregates 3:1, allowing a reduction of 25% in seeded offspring, or 50% considering that homozygous seedless offspring might have an undesirable berry size due to the total lack of hormones from seeds. In agricultural practice, the identification and discard of seeded offspring permits reduction of the costs, space, and time required to develop new varieties by up to 50%, making breeding more efficient and more environmentally friendly.

Taking into consideration the above-mentioned disadvantages in the use of p3_VvAGL11 SSR, we re-designed the marker, now called 5U_VviAGL11, which resulted in a more informative, precise, and reliable marker (Figure 5 and Figure 6). In a *Vitis vinifera* background, the SSR 5U_VviAGL11 has a polymorphism information content (PIC) value very close to 0.9 (it reveals up to 19 alleles in *Vitis vinifera* and five additional alleles were identified in other *Vitis* species (Figure 6 and Supplementary Tables S5 and S6) and could be used as a standard marker for varietal identification, in addition to the phenotype prediction.

Furthermore, considering that *VviAGL11* explains less than 50% of the phenotypic variation, which results in significant false negative and positive rates, and that the mutation (Arg-197-Leu, [G/T] SNP) from the coding region was identified as causal [16], we considered a shift to an SNP-based assisted selection to maximize accuracy. For routine assisted selection, we developed two SNP-based markers that identify the causal Arg-197-Leu mutation ([G/T] SNP), the first of which is based on qPCR-HRM (Figure 7) and the second of which is based on Tetra-ARMS-PCR (Figure 8). If marker assays are not cost-effective and fast in screening routines, they have a limited impact. Thus, to make assisted selection realistic—which includes an ability to genotype thousands of genotypes in a short span of time, i.e., 3–4 months, when the objective is to plant seedlings derived from the previous year’s crosses in the field—we coupled e7_VviAGL11 genotyping by qPCR-HRM to a fast and non-expensive DNA extraction protocol suitable for grapevine leaf samples. The protocol originally described by Xin et al. [27], slightly modified from its original version, was directly applied to grapevine genotyping.

The rate of false negative and positives was zero when we used the causal mutation (Arg-197-Leu) SNP as a tool for assisted selection in a large pool of varieties (*n* = 209, Tables S5 and S7). However, this perfect association might be biased because, as varieties, these individuals passed already through a selection process that drives the seedless phenotype to smaller values. Nevertheless, the screening of varieties allowed us to identify 10 varieties that presented evidence of a recombination event between the regions evaluated by SSRs p3_VvAGL11 or 5U_VviAGL11 in the 5′UTR and exon 7 from the coding region evaluated by the SNP e7_VviAGL11 (Supplementary Table S5). The association between the seedless 5’UTR region and the seeded exon 7 from the coding region could also be due to the existence of an ancestral seeded allele, such as one originating from a seeded Sultanina, e.g., Sultanina Monococco (Supplementary Table S5). Among these individuals, two were derived from CHAOUCH BLANC: DUC DE MAGENTA (CHASSELAS × (CHAOUCH BLANC × KOKUR KRASNYI)) and RANNI VIRA (CHAOUCH BLANC × KISHMISH CHERNYI), suggesting that CHAOUCH BLANC is a possible source of a recombination that disables the tracking of the seedless phenotype with SSRs p3_VviAGL11 or 5U_VviAGL11. This origin of false positive varieties was also suggested by Karastan et al. [100]. Additionally, two other recombinant individuals were found to be descendants of CORNICHON BLANC: ORLOVI NOKTI BELI (PARMAK CERVEN × CORNICHON BLANC) and PIZZUTELLO NERO (CORNICHON BLANC × PRUNE DE CAZOULS), indicating that Cornichon Blanc might also be a source of false positive associations. This last one was not previously reported. At the moment, it is not possible to trace if Cornichon Blanc and Chaouch Blanc share a common origin. These results also support the use of SNP e7_VviAGL11 for assisted selection given that it is based on causal mutation. Within the MA × CS progeny (*n* = 573), we identified two (0.35%) individuals (a seeded promoter with a seedless coding region and a seedless promoter with a seeded coding region) that indicate evidence for intragenic recombination, reflecting that such events are not extremely rare, contributing to a reduction in the accuracy of markers based on the 5′UTR region of *VviAGL11*. 

For germplasm or parental characterization, we propose the combined use of 5U_VviAGL11 SSR and e7_VviAGL11 SNP together. For routine selection, the e7_VviAGL11 SNP could improve efficiency (simplifying the genotyping process, decreasing genotyping costs, and increasing the efficiency of the overall process). Since the cost of equipment, supplies, and trained personnel is variable between breeding programs and countries, we compared the principal benefits and requirements for all developed markers (Supplementary Table S12) so that potential new users can choose which one best suits their expectations and requirements.

## 5. Conclusions

From a historical perspective, the hypothesis proposed by Bouquet and Danglot [10] regarding the inheritance of seedlessness controlled by a major dominant locus and three independent minor loci still seems to be valid, and the genetic architecture reveals that seedlessness is a complex trait controlled by the *VviAGL11* gene as the dominant major locus and by up to 11 stable and small-effect QTLs. For these QTLs, we proposed a list of 74 candidate genes that might contribute to the understanding of the fine control of the inheritance of seedlessness. Besides *VviAGL11*, which controls ovule and seed development, other interesting genes involved in sex flower determination, flowering time, and probably the number of seeds are a gene member of the *YABYY* family, *INP1* and *APT3* For *YABBY*, an intragenic SSR already exists (VVIB23) and can be tested to improve assisted selection for the number of seeds. 

The identified associations are valid in the MA × CS progeny, and they need to be validated in other progenies in order to identify the most favorable alleles for breeding before the development of markers for assisted selection. Experiments might be tough due to small-effect QTLs that require large populations for validation purposes, and for this, the breeding community might help to coordinate such efforts. We tested a multi-QTL assisted selection strategy that has improved accuracy when favorable alleles are selected together, allowing for multiloci assisted selection after QTLs validation. This strategy which might boost breeding efficiency based on molecular selection for complex traits.

For assisted selection, we improved upon the results of using an SSR-based marker located in the 5′UTR of *VviAGL11*, establishing 5U_VviAGL11 as a replacement for p3_VvAGL11, or to be used in combination to reduce the costs of co-electrophoresis. However, it is important to be aware that recombination events between the 5′UTR and the causal mutation located in the coding sequence breaks the association and the ability to predict might then be lost. To track seedlessness by causal mutation, we developed two methodologies, qPCR-HRM and Tetra-ARMS PCR, that make routine selection simpler, faster, as well as more accurate and cost-efficient.

## Figures and Tables

**Table 1:** QTLs detected for seedlessness in the MA × CS population during four seasons.

Trait	Chrom.	Position (cM)	SNP Cofactor	LOD	% Expl. Var	Mean Genotype a	Mean Genotype b	LOD				Stable	QTL	Support
								2015	2016	2017	2018			
**SEDW**	9MA	33.108	chr9_5511355_1	2.26	1.7	0.0867	0.0753	0.98	0.93	0.72	3.28	No	Significant	BLUP + 1 Season
	13MA	1.345	chr13_810814_1	1.96	1.5	0.0740	0.0870	1.99	0.89	1.54	1.93	Yes	Suggestive	BLUP + 2 Seasons
	13MA	61.553	chr13_14817494_1	2.39	1.8	0.0742	0.0886	0.53	1.5	2.5	1.88	Yes	Suggestive	BLUP + 2 Seasons
	4CS	63.423	chr4_19816331_1	2.33	0.9	0.0885	0.0740	2.24	1.03	1.78	1.47	Yes	Suggestive	BLUP + 2 Seasons
	14CS	10.51	chr14_3367810_1	2.06	0.8	0.0758	0.0880	0.19	1.02	0.93	1.49	No	Suggestive	BLUP only
	18CS	96.474	e7_VviAGL11	90.4	48.4	0.1052	0.0410	38.85	73.61	58.93	46.78	Yes	Significant	BLUP + 4 seasons
**SEDN**	1MA	23.448	chr1_4720746_1	4.03	2.8	2.8760	3.2148	1.95	2.85	0.88	1.91	Yes	Significant	BLUP + 3 seasons
	2MA	31.857	chr2_5144635_1	12.81	9.2	2.6574	3.3845	5.41	6.59	0.73	16.5	Yes	Significant	BLUP + 3 seasons
	8MA	53.979	chr8_16567159_1	2.06	1.4	3.2588	2.9028	1.5	0.74	0.89	0.64	No	Suggestive	BLUP only
	13MA	1.345	chr13_810814_1	2.49	1.7	2.8896	3.1877	1.19	0.74	0.24	2.03	No	Suggestive	BLUP + 1 Season
	1CS	68.878	chr1_20882467_1	2.82	1.9	3.2313	2.8568	0.24	4.49	0.59	0.85	No	Suggestive	BLUP + 1 Season
	2CS	30.109	chr2_5443648_1	2.48	1.6	3.2588	2.9172	1.61	0.36	1.3	3.7	No	Suggestive	BLUP + 1 Season
	7CS	99.657	chr7_24375532_1	2.46	1.6	3.1993	2.8989	0.79	0.98	0.13	2.97	No	Suggestive	BLUP + 1 Season
	12CS	4.672	chr12_1741069_1	2.95	1.9	3.2576	2.8558	0.94	3.43	2.35	0.43	Yes	Suggestive	BLUP + 2 Seasons
	12CS	65.968	chr12_22397742_1	2.76	1.8	2.8865	3.2123	2.02	1.43	0.35	1.73	Yes	Suggestive	BLUP + 2 Seasons
	18CS	96.474	e7_VviAGL11	5.27	3.5	3.2574	2.7010	3.51	1.87	0.61	4.84	Yes	Significant	BLUP + 2 Seasons
**SEDA**	2MA	32.691	chr2_5330875_1	4.32	3.2	0.0797	0.0968	0.25	3.57	0.18	8.96	Yes	Significant	BLUP + 2 Seasons
	9MA	8.439	chr9_1387529_1	2.56	1.9	0.0815	0.0944	2.28	1.10	0.72	2.24	Yes	Suggestive	BLUP + 2 Seasons
	13MA	61.22	chr13_14790761_1	2.27	1.7	0.0938	0.0826	0.13	2.15	5.15	1.06	Yes	Suggestive	BLUP + 2 Seasons
	2CS	27.274	chr2_5222338_1	3.95	1.6	0.0788	0.0933	0.74	0.09	1.43	4.42	No	Significant	BLUP + 1 Season
	8CS	50.684	chr8_17020635_1	2.66	1.1	0.0878	0.0972	0.02	1.79	1.92	2.52	Yes	Suggestive	BLUP + 3 seasons
	10CS	54.671	chr10_8168185_1	3.15	1.3	0.0827	0.0827	1.16	0.92	0.59	2.89	No	Significant	BLUP + 1 Season
	14CS	10.51	chr14_3367810_1	1.93	0.8	0.0878	0.0834	0.33	0.48	1.22	0.89	No	Suggestive	BLUP only
	18CS	96.474	e7_VviAGL11	76.08	42.5	0.1107	0.0506	20.74	43.37	53.98	38.99	Yes	Significant	BLUP + 4 seasons

For each analyzed seedless subtrait, SEDW: seed fresh weight per berry; SEDN: number of seeds per berry; SEDA: average fresh weight of one seed. QTLs are detailed in the respective chromosomes for each parental genotype (MA or CS). Logarithm of the odds (LOD) and percent of variation explained (% Var. Expl.) were calculated by multiple QTL mapping (MQM) analysis using cofactors defined in the fourth column after preliminary interval mapping, Kruskal–Wallis, and automatic cofactor selection analysis. A QTL is declared if it is detected by MQM analysis (at suggestive or significant level) using BLUP values, and it is called stable if it is detected independently at least for two of the four analyzed seasons. The effect of the a or b genotypes, corresponding to the homozygous <aa> or heterozygous <ab> genotype respectively, are indicated as the mean phenotypic value for SEDW, SEDN, and SEDA. SNPs segregating from Muscat of Alexandria have the genotype <ab × aa>, and those from Crimson Seedless have the genotype <aa × ab>.

**Table 2:** Evaluation of a multiloci assisted selection scheme in the MA × CS experimental progeny.

**Multiloci Assisted Selection in Bi-Parental Population, One-Way ANOVA Model Summary for SEDW, *n* = 571**												
**Order by % Var. Expl.**	**Parental**	**QTL Cofactor**	**Individual Favorable Genotype**	**Nº of QTLs**	**Factors**	**Cumulative Favorable Genotype (Ideotype)**	**Ideotype N**	**Ideotype Mean (g)**	**Ideotype Std. Dev.**	**Ideotype 95% C.I.**	**R² % (Adjusted)**	***p*-Value**
1	CS	e7_VviAGL11	b	1	2	b	217	0.0412	0.0256	(0.03776;0.04462)	52.11%	0.000
2	MA	chr13_14817494_1	a	2	4	ba	122	0.0363	0.0233	(0.03208;0.04045)	53.71%	0.000
3	MA	chr9_5511355_1	b	3	8	bab	70	0.0321	0.0184	(0.02773;0.03653)	54.38%	0.000
4	MA	chr13_810814_1	a	4	16	baba	43	0.0316	0.0213	(0.02505;0.03815)	55.41%	0.000
5	CS	chr4_19816331_1	b	5	32	babab	29	0.0308	0.0234	(0.02187;0.03968)	55.98%	0.000
**Multiloci Assisted Selection in Bi-Parental Population, One-Way ANOVA Model Summary for SEDN, *n* = 572**												
**Order by % Var. Expl.**	**Parental**	**QTL Cofactor**	**Individual Favorable Genotype**	**Nº of QTLs**	**Factors**	**Cumulative Favorable Genotype (Ideotype)**	**Ideotype N**	**Ideotype Mean (number)**	**Ideotype Std. Dev.**	**Ideotype 95% C.I.**	**R² % (Adjusted)**	***p*-Value**
1	MA	chr2_5144635_1	a	1	2	a	267	2.6574	1.1862	(2.5145;2.8003)	9.15%	0.000
2	CS	e7_VviAGL11	b	2	4	ab	100	2.4220	1.0830	(2.208;2.637)	14.63%	0.000
3	MA	chr1_4720746_1	a	3	8	aba	44	2.1980	1.0930	(1.866;2.530)	17.09%	0.000
4	CS	chr1_20882467_1	b	4	16	abab	19	1.8610	0.9310	(1.412;2.310)	19.46%	0.000
5	CS	chr12_1741069_1	b	5	32	ababb	13	1.7950	0.8360	(1.289;2.300)	21.72%	0.000
**Multiloci Assisted Selection in Bi-Parental Population, One-Way ANOVA Model Summary for SEDA, *n* = 569**												
**Order by % Var. Expl.**	**Parental**	**QTL Cofactor**	**Individual Favorable Genotype**	**Nº of QTLs**	**Factors**	**Cumulative Favorable Genotype (Ideotype)**	**Ideotype N**	**Ideotype Mean (g)**	**Ideotype Std. Dev.**	**Ideotype 95% C.I.**	**R² % (Adjusted)**	***p*-Value**
1	CS	e7_VviAGL11	b	1	2	b	218	0.05056	0.02813	(0.04681;0.05432)	43.87%	0.000
2	MA	chr2_5330875_1	a	2	4	ba	119	0.05004	0.02631	(0.04526;0.05481)	48.83%	0.000
3	MA	chr9_1387529_1	a	3	8	baa	71	0.04831	0.02505	(0.04238;0.05424)	49.06%	0.000
4	MA	chr13_14790761_1	b	4	16	baab	34	0.03942	0.01989	(0.03248;0.04636)	49.82%	0.000
5	CS	chr2_5222338_1	a	5	32	baaba	15	0.03227	0.01562	(0.02362;0.04092)	51.92%	0.000

For each analyzed seedless subtrait: SEDW: seed fresh weight per berry; SEDN: number of seeds per berry; SEDA: average fresh weight of one seed. A multiloci model was created considering the five SNPs that have the largest contribution in terms of the individual variance that they explain in the MQM analysis (Table 1). The addition of markers in decreasing order for their individual explained variance and the selection of the individual favorable genotype creates an ideotype for whom the number of individuals (Ideotype N) is indicated, as well as the phenotype (Ideotype Mean) and its 95% confidence interval (95% C.I.). The model was evaluated by a one-way ANOVA considering all the possible haplotypes (Factors). The adjusted R-squared (R^2^ %) that represents the proportion of the variance explained by the model and *p*-value are also indicated. The model includes markers derived from both parental genotypes (MA or CS).

**Table 3:** Summary of analyzed material evaluated for validation assays.

Type of Material	Detail of Material	Number of Genotypes	p3_VvAGL11 SSR	U_VviAGL11 SSR	e7_VviAGL11 SNP
Variety collection	Seeded Varieties	161	151 Seeded and 10 Seedless	151 Seeded and 10 Seedless	161 Seeded [G/G]
Variety collection	Seedless Varieties	48	48 Seedless	48 Seedless	48 Seedless [G/T]
Experimental genotype	Control homozygous seedless	2	2 Seedless	2 Seedless	2 Seedless [T/T]
F1 progeny	Seeded Progeny (RG × AR)	31	30 Seeded and 1 Seedless	30 Seeded and 1 Seedless	30 Seeded [G/G] and 1 Seedless [G/T]
F1 progeny	Seedless Progeny (RG × AR)	17	4 Seeded and 13 Seedless	4 Seeded and 13 Seedless	4 Seeded [G/G] and 13 Seedless [G/T]
F1 progeny	Seeded (RG × RS)	18	16 Seeded, 1 Seedless and 1 NA	17 Seeded and 1 Seedless	17 Seeded [G/G] and 1 Seedless [G/T]
F1 progeny	Seedless (RG × RS)	30	1 Seeded and 29 Seedless	1 Seeded and 29 Seedless	1 Seeded [G/G] and 29 Seedless [G/T]
Breeding material	Seeded Progeny (multiple crosses)	56	54 Seeded and 2 Seedless	54 Seeded and 2 Seedless	54 Seeded [G/G] and 2 Seedless [G/T]
Breeding material	Seedless Progeny (multiple crosses)	118	110 Seedless and 8 NA	118 Seedless	118 Seedless [G/T]
Other *Vitis* species	Other *Vitis*	9	9 Seeded	9 Seeded	8 Seeded [G/G]
Other Vitaceae members	Other Vitaceae	4	4 Seeded	4 NA	5 Seeded [G/G]

## Supplementary Materials

The following are available online at https://www.mdpi.com/2073-4425/11/2/151/s1. Figure S1: Alignment and dendrogram of 5′UTR region of *VviAGL11* gene for the design of a new SSR (short sequence repeat) 5U_VviAGL11 for assisted selection. Figure S2: Design and location of specific primers for the identification of Arg-197-Leu mutation in VviAGL11 by qPCR-HRM with primers e7_VviAGL11 and by Tetra-ARMS PCR. Figure S3: Histograms and trend lines assuming a 3-parameter loglogistic distribution. Figure S4: Spearman’s correlation between SEDW, SEDN, and SEDA during 2015, 2016, 2017, and 2018. Figure S5: Whole genome QTL detection for seedlessness subtraits SEDW, SEDN, and SEDA in the MA × CS experimental population. Figure S6: qPCR-high resolution melting analysis for the detection of the SNP [G/T] with an e7_VviAGL11 marker. Figure S7: Air temperature during flowering time (from November 5 to 18) across four evaluated seasons (2015-2018) at La Platina Regional Research Center where the MA × CS population is maintained. Table S1: Details of primer pairs developed to identify Arg-197-Leu mutation, in the *VviAGL11* gene responsible for the seedless phenotype, by qPCR-HRM analysis and Tetra-ARMS-PCR. Table S2: Genetic parental maps construction results details. Table S3: Genes mapped in reference genome Pinot Noir PN40024 within QTLs confidence intervals for SEDW, SEDN and SEDA identified in the MA × CS experimental progeny Genes. Table S4: Candidate genes within QTLs confidence intervals for SEDW, SEDN, and SEDA identified in the MA × CS experimental progeny. Table S5: Analyzed material evaluated for validation assays. Table S6: Heterozygosity and polymorphism information content of p3_VvAGL11 and 5U_VviAGL11 SSR markers evaluated in a *Vitis vinifera* background. Table S7: Contingency table to evaluate the prediction performance of developed markers in varieties. Table S8: Contingency table to evaluate the prediction performance of developed markers in the Red Globe × Autumn Royal progeny. Table S9: Contingency table to evaluate the prediction performance of developed markers in the Red Globe × Regal Seedless progeny. Table S10: Contingency table to evaluate the prediction performance of developed markers in segregants from multiple progenies. Table S11: Contingency table to evaluate the prediction performance of developed markers in the Muscat of Alexandria × Crimson Seedless progeny. Table S12: Considerations for the implementation of assisted selection based on the use of SSR 5U_VviAGL11 or SNP e7_VviAGL11 revealed by qPCR-HRM or Tetra-ARMS-PCR.
